# Power of Lupulin in Allaying Irritation of the Genital Organs

**Published:** 1850-11

**Authors:** George Zeigler

**Affiliations:** Philadelphia


*Power of Lupulin in allaying Irritation of the Genital Organs.* By GEORGE ZEIGLER, M. D., of Philadelphia. Being obliged to resort to a prolonged use of tonics, I took the combination of iron, sulphate of quinia, and lupulin. After some time had elapsed, I believed that the indication for which I took lupulin was fulfilled. I therefore omitted it, still continuing the use of iron and the quinia, ½ grain of the latter three times a day. In a few days, I began to experience symptoms of irritation of the bladder and urethra, with pain at the extremity of the penis, and frequent desire for micturition, somewhat similar to those resulting from calculus. These symptoms increased, and in two or three weeks became so violent and constant as to give me no peace. The thought then occurred that they might result from something I was taking, and my mind reverted to the possibility of their proceeding from the quinia. This idea was confirmed by the opinion of a medical friend. As the symptoms did not appear during the use of the combination of lupulin with the other remedies, we concluded that it would be better to recombine them. This was done, and with the happiest effect. The feelings of distress began immediately to subside, and in a few weeks entirely disappeared; and although I have continued the use of the above mentioned remedies for months since, I have had no further symptoms of the kind.—*Am. Jour. Med. Sci.*

